# Invisible burdens of platform work: a qualitative study of food-delivery riders’ lived experiences in urban India

**DOI:** 10.1080/17482631.2026.2644577

**Published:** 2026-03-14

**Authors:** Ashifa Kariveliparambil, Rasi R A, Muhammad Shakil Ahmad, Snega Ramesh, Alen Kuriakose

**Affiliations:** a School of Humanities and Arts, Xi’an International University, Xi'an, Shaanxi, People's Republic of China; b Faculty of Health Sciences, Istanbul Gelisim University, Istanbul, Türkiye; c Department of Social Sciences, School of Social Sciences and Languages, Vellore Institute of Technology, Vellore, India; d Teesside University International Business School, Teesside University, Middlesbrough, United Kingdom; e Faculty of Business, Sohar University, Sohar, Oman; f Széchenyi István University, Egyetem Square 1, Győr, Hungary; g School of Social Work, Marian College Kuttikkanam (Autonomous), Kuttikkanam, India

**Keywords:** Gig economy, food-delivery riders, labour precarity, emotional labour, symbolic violence, psychosocial health, reflective thematic analysis

## Abstract

**Purpose:**

This study explored the lived experiences of food delivery riders in India’s platform economy, focusing on psychosocial, physical, and emotional challenges embedded in their daily work. It critically examines how precarity, emotional labor, and symbolic violence shape riders’ well-being.

**Methods:**

This study employed a qualitative phenomenological design. In-depth interviews were conducted with 10 food delivery riders from an urban district in South India, who participated anonymously. The data were analyzed using reflexive thematic analysis.

**Results:**

Surviving precision, embodied exhaustion, emotional discipline under surveillance, internalized struggles, and fragmented routines emerged as key themes. Participants described working under difficult climatic conditions and persistent physical pain, reflecting the precarious nature of platform-based delivery work. They also struggled to maintain constant politeness with customers while being monitored through algorithmic surveillance. This findings reveal significant psychosocial burdens, including physical strain, emotional fatigue, social withdrawal, experience of disrespect, internalization of blame, and economic insecurity.

**Conclusions:**

Platform work reinforces structural precarity, emotional suppression, and symbolic exclusion, profoundly shaping the health and well-being of food delivery workers. These findings highlight the need for policy reforms to protect gig workers’ mental health, dignity, and social inclusion, and advocating for the psychosocially sensitive governance of digital labor platforms.

## Introduction

The Indian food delivery sector has seen remarkable growth, with market revenue expected to exceed $54 billion by 2025 (Statista, 2025). This surge is fuelled by rapid digitalisation, which has increased smartphone adoption and changed urban lifestyles (Lanfranchi et al., 2025). Every day, thousands of delivery riders navigate crowded urban centres, and their work is governed by the invisible algorithms of Zomato, Swiggy, and Uber Eats (Rani & Dhir, 2020; Yu et al., 2022). These workers play a vital yet largely invisible role, carrying out time-sensitive, inconsistent earnings, physically demanding tasks, and constantly threatening deactivation (Komarraju et al., 2021; Popan & Anaya-Boig, 2022). Although the gig economy is often praised for its autonomy and low barriers to entry, a growing body of international research has painted a sobering picture. Studies have increasingly revealed that such work is defined not by freedom but by relentless precision, emotional strain, symbolic subordination, and negative health effects (Wood et al., 2018).

Food delivery workers are increasingly being positioned at the intersection of structural insecurity and digital control. Existing research on platform labour underscores that gig workers routinely operate without employment contracts, social protections, or stable income, and are subject to opaque, asymmetrical decision-making by platform algorithms (Chan, 2022; Sharma, 2020). These conditions create a structurally embedded precarity, defined not only by material insecurity but also by temporal unpredictability, the need to self-finance work tools (vehicles, fuel, and mobile data), and constant availability (Vallas & Schor, 2020). Recent labour process research further shows that piece-rate payment systems in platform delivery function as indirect modalities of control, disciplining workers’ time, pace, and movement while individualising risk (De Krijger, 2023). In India, these challenges are further exacerbated by the informal nature of the labour market, limited regulatory oversight, and the normalisation of under-employment (Laskar, 2023). Empirical studies show that Indian delivery riders often work 10–12 hours daily yet earn wages below the minimum wage (Krishna, 2020; Mehta, 2020). The economic fragility of their work is compounded by their vulnerability to accidents, exploitation by vendors, customer harassment, and a complete absence of social insurance (Huang, 2021; Popan & Anaya-Boig, 2022).

The consequences of precarious work arrangements on workers’ health and well-being are significant. Strong public health literature documents precarious employment as an important social determinant of health, associated with a higher risk of mental distress, anxiety, musculoskeletal disorders, and burnout (Benach et al., 2014; Jalil et al., 2023). For gig workers, these risks are intensified by the invisibility of their labour, the absence of collective representation, and the psychological toll of constant surveillance and customer dependency (Lang et al., 2023). In India, infrastructural regulatory gaps further amplify these risks, and the situation is becoming a growing public health concern.

Beyond economic and physical precarity, the emotional dimensions of gig work have become increasingly salient in contemporary research. Hochschild’s concept of emotional labour (Hochschild, 2015)—the management of feelings to comply with occupational expectations–has gained relevance in the platform economy, in which customer-facing metrics govern worker success (Bucher et al., 2020; Wharton, 2009). Ratings-based accountability induces stress and emotional self-regulation because negative reviews can lead to reduced visibility, job loss, and bonuses (Rosenblat and Stark, 2016). These expectations transform customer interactions into psychological strain and self-discipline. In India, these emotional pressures intersect with caste- and class-based hierarchies, intensifying riders’ sense of vulnerability (Krishna, 2020; Vijaya Sankari & Kalpana, 2021). The persistence of such self-blame and the normalisation of hardship reflect what Bourdieu terms symbolic violence (Bourdieu, 1991), where structural inequalities are internalised as individual failures.

Despite a growing body of literature on gig work, research on these lived experiences remains limited. Few Indian studies have explored how delivery riders interpret and internalise their psychosocial struggles within the platform economy (Parwez & Ranjan, 2021). The intersection of structural, emotional, and symbolic forces shaping health and well-being remains critically under-explored, particularly in psychosocial dimensions such as anxiety, emotional regulation, internalised guilt, and social disconnection (Wood et al., 2018). Understanding these processes is crucial for developing context-sensitive insights into how digital labour affects identity, dignity, and health.

This study responds to this need through a phenomenological exploration of the lived experiences of food delivery riders in Southern India. This advances the existing research in three ways. First, it integrated the structural, emotional, and symbolic dimensions of gig work to provide a holistic understanding of precarity. Second, it foregrounds riders’ narratives, revealing how agency and endurance coexist with exhaustion and indivisibility. Third, it contributes theoretically by linking emotional labour and symbolic violence to a distinct form of precarious platform that characterises the Indian gig economy. This study aimed to address the following core research questions:

1. How do food delivery riders in India experience and interpret the emotional, physical, and social challenges embedded in their daily interactions?
2. How do structural precarity, emotional strain, and symbolic exclusion manifest in the lived experiences of food delivery riders, and how do they shape their overall well-being?

## Literature review and analytical framework

A growing body of scholarship from the wider Asian region has also interrogated the precarious realities of platform-based gig work, revealing both commonalities and divergences with the Indian experience. Studies from Malaysia have highlighted the urgent need for worker-centred policies to strengthen social protection and reduce inequality (Abd Samad et al., 2023). In Indonesia, researchers have documented hidden managerial controls exerted by platforms through cost deductions and performance pressure, often masked by the rhetoric of flexibility (Putri et al., 2023; Wulansari et al., 2024; Yasih, 2023). In China, the absence of formal employment contracts has been linked to inadequate occupational coverage, leaving workers vulnerable to income and health shocks (Stuart et al., 2023). Gender disparities also persist; female workers face stigma, technological barriers, and heightened emotional strain (Bishwakarma et al., 2024; Kwan, 2024). In South Korea, gig workers endure verbal and sexual harassment, and such exposure is linked to depression (Kim et al., 2023). Quality-of-life studies similarly underline the emotional and environmental pressures that shape workers’ wellbeing (Kim et al., 2023). Collectively, these findings situate India’s food delivery workforce within a broader regional landscape of precarious, algorithmically mediated labour, while reinforcing the need for nuanced, context-sensitive enquiry. Similarly, research from other Asian urban contexts demonstrates that platform-based couriers experience cumulative hassles, time pressure, and psychological strain shaped by regulatory gaps and customer dependency (Zong et al., 2024), reinforcing the regional embeddedness of such vulnerabilities.

While global scholarship offers extensive insights into the dynamics of platform labour, recent South Asian research offers contextually grounded perspectives. In India, studies demonstrate that food delivery work intersects with entrenched hierarchies of caste, class, and gender, shaping both access to and experience within gig employment (Gupta et al., 2022; Komarraju et al., 2021). Women’s participation remains limited due to safety concerns and mobility constraints (Gupta, 2020; Singh, 2023), while caste-based occupational segregation often channels marginalised groups into the most precarious delivery roles. Similar trends in informal recruitment and weak regulations in Nepal and Bangladesh have exacerbated insecurity (Hamal & Huijsmans, 2022; Hsu, 2025). These patterns highlight that the vulnerabilities of the gig economy in the Global South are embedded in historical social stratifications and gendered exclusions, making intersectionality an essential analytical lens. Such conditions, widely reported in Indian cities, exemplify the convergence of material and affective precarity and the environment in which workers endure physical hardship and emotional depletion, despite institutional support.

This study is grounded in three interrelated theoretical perspectives: *emotional labour* (Hochschild, 2015), *symbolic violence* (Bourdieu, 1991), and *platform precariousness* (Huang, 2021), which collectively offer a critical lens for interrogating the lived experiences of India’s food delivery workers. These frameworks not only inform the interpretation of findings but also guide the design of the interview protocol, ensuring that the research questions explicitly capture the structural, emotional, and symbolic dimensions of gig work.

### Emotional labour

Hochschild’s concept (Hochschild, 2015) refers to the regulation of emotions to meet occupational expectations. In platform-mediated work, riders must display patience and politeness regardless of personal strain or customer hostility (Bucher et al., 2020; Wharton, 2009). Ratings-based accountability systems amplify this demand by linking affective performance to income and access (Rosenblat and Stark, 2016). This framework captures how digital platforms commodify emotional expressions, transforming courtesy into a survival strategy under algorithmic surveillance.

### Symbolic violence

Bourdieu’s notion of symbolic violence (Bourdieu, 1991) explains how inequality becomes internalised as natural or deserved. In a gig economy, it manifests in the acceptance of exploitation—long hours, unsafe conditions, and a lack of benefits–as self-chosen or unavoidable (Cruz & Gameiro, 2023). Workers may attribute missed bonuses or delivery delays to personal shortcomings rather than systemic failures, reinforcing their own subordination. The interview questions informed by this framework examined perceptions of supervisory treatment, restaurant staff interactions, denial of rest or essential needs, and the internalisation of blame for factors beyond riders’ control. Platform rhetoric that glorifies ‘hustle culture’ and self-reliance reinforces this misrecognition, encouraging workers to interpret hardship as resilience rather than systemic neglect (Newlands, 2020).

### Platform precariousness

This concept denotes the instability of gig work shaped by algorithmic management, fluctuating demand, and the absence of regulations (Vallas & Schor, 2020; Wood et al., 2018). It encompasses irregular income, unpredictable working hours, exposure to hazardous conditions, and the financial burden of self-provisioning work tools (Fairwork India, 2024). This framework contextualises riders’ economic fragility, exposure to unsafe environments, and dependence on opaque digital rating systems as structural phenomena. Platform delivery has also been conceptualised as a form of territorial labour, where riders’ agency is negotiated within spatially constrained urban logistics networks shaped by algorithmic governance (Miszczynski & Pieczka, 2024).

By integrating these perspectives, the analytical framework positions gig as a nexus between affective regulation, internalised subordination, and economic instability. This teiadic lens allows the study to examine how the interplay between emotional labour, symbolic violence, and platform precariousness shapes workers’ psychosocial well-being. It also clarifies the theoretical grounding for interpreting riders’ narratives as reflections of both agency and domination within digitally mediated labour regimes.

## Materials and methods

This qualitative study was situated within a constructivist–interpretivist paradigm, recognising that reality is socially constructed and best understood through individuals’ subjective lived experiences. Given the focus on the embodied meanings and challenges of food delivery in Southern India, phenomenology was adopted as a guiding methodological approach. It prioritises contextual depth over statistical generalisability (Priya, 2017). Reflexive thematic analysis (Braun & Clarke, 2019) provides a structured, yet flexible framework for interpreting complex psychosocial realities. Recognising that knowledge is co-constructed, this study emphasises the cultural and linguistic contexts. The native Tamil-speaking researcher conducted all the interviews to ensure rapport and authenticity.

### Participants and recruitment

Participants were recruited from an urban district in Southern India through purposive and snowball sampling. Eligibility required at least six months of active food delivery experience and willingness to participate in an in-depth interview. Both full-time and part-time riders were included in the study. Interviews were arranged during the riders’ scheduled breaks to accommodate irregular work patterns.

Ten participants, aged 24–35 years, participated in this study. This reflects the demographic profile of the Indian delivery sector, which is dominated by younger workers performing physically demanding, unstable labour (Kavitha & Parvathy, 2023; Sarker et al., 2024). Nine were identified as male and one as female, mirroring national gender distributions rather than researcher selection bias (Bishwakarma et al., 2024; Kwan, 2024). Most came from low-income to middle-income households, with several rural migrants seeking urban employment. Although religion and caste were included in the demographic questions, some participants declined to respond, and these variables were excluded from the analysis. A sample size of 10 was determined based on saturation, in which no new themes emerged (Hennink & Kaiser, 2022).

### Data collection and analysis

Data were collected between June and August 2024 through in-depth, semi-structured, face–to–face interviews in Tamil. The flexible question guide elicited narratives on occupational stress, emotional strain, health, and perceptions of dignity. Each interview lasted 60–90 min and was audio-recorded with verbal consent. Recordings were transcribed verbatim and translated into English by bilingual experts, and translations were verified collaboratively to preserve contextual nuances.

Data were analysed using Braun and Clarke’s six-phase reflexive thematic analysis (Braun & Clarke, 2019), which offers a flexible yet systematic approach to interpreting participants' rich experience-based narratives. The analysis aimed to capture both explicit patterns and deeper interpretive meanings within participants’ accounts while maintaining a reflexive awareness of the researchers’ assumptions (Tufford & Newman, 2012).

The research team began with an immersive familiarisation process, repeatedly reading transcripts and reviewing audio recordings to ensure accuracy and contextual sensitivity. Initial codes were generated inductively, allowing categories to emerge organically from the data without being constrained by pre-existing theoretical constructs. Codes were applied to phrases, incidents, and emotional expressions that reflected psychological distress, physical burden, financial strain, and perceptions of stigma. These codes were then collated into preliminary thematic clusters and reviewed collaboratively to enhance the analytic coherence and depth. Through this iterative process, five core themes were developed that captured the recurring structures and emotional undercurrents in participants’ accounts: *surviving precision, embodied exhaustion, emotional discipline under surveillance, internalised struggles, and fragmented routine and physical deterioration*. NVivo 12 software facilitates data management and code refinement. Reflexive journaling and peer discussions supported the analytic consistency. The final themes evolved through phenomenological interpretation, progressing from significant to overarching thematic essences.

### Ethical considerations

This study was conducted in accordance with the ethical principles outlined in the Declaration of Helsinki. This study was approved by the ethics committee of the Vellore Institute of Technology, India (Approval Number: 2024/22MTW0025). Participants were fully informed about the study’s objectives, voluntary participation, and their right to withdraw at any stage without penalties. Verbal informed consent was obtained and audio recorded prior to each interview in accordance with the participants’ preferences for minimal formal procedures, considering their occupational time constraints and the need to ensure comfort and openness during the interviews. Confidentiality, anonymity, and respect for participants' autonomy were strictly maintained throughout the research process.

### Trustworthiness and reflexivity

To ensure methodological rigour, this study adhered to Lincoln and Guba’s (1985) criteria for trustworthiness: credibility, transferability, dependability, and confirmability (Lincoln & Guba, 1985). The interviewers and participants spoke the same language; constant engagement and member-informed transcription helped establish credibility. Transferability was supported by providing thick descriptions of the participants' contexts and experiences, as well as the research context. To maintain dependability, an audit trail of coding processes, theme development, and analytical decisions was conducted. Reflexive journaling, peer debriefing with the research team, and regular analytical discussions were conducted to enhance the study’s confirmability. Furthermore, it helped lessen individual bias, and the participants’ text supported the findings. Reflexivity was a continuous process throughout the study. The researchers remained critically aware of their own positionalities, particularly in terms of language, class, and educational background, and how they could shape both data interpretation and meaning construction. The involvement of culturally and linguistically embedded interviewers plays a central role in ensuring authenticity, reducing hierarchical distance, and deepening interpretive richness.

## Results

This section presents the findings derived from in-depth interviews with ten food delivery riders working for major platform companies in urban India. Five core themes were constructed using reflexive thematic analysis to capture the psychosocial, emotional, and physical dimensions of their work experience. The analysis aimed not only to describe surface-level issues but also to interpret how riders understood, internalised, and coped with the structural, symbolic, and affective demands of platform-based labour. The findings are organised into two parts. The first subsection outlines the participants' socio-demographic profiles to contextualise their narratives. The second subsection introduces five interpretive themes, each reflecting recurring patterns of meaning grounded in participants’ accounts. Where appropriate, direct quotations were used to preserve participants' voices and illuminate the subjective weight of their experiences. The themes are interpreted within the conceptual framework of labour precarity, emotional labour, and symbolic violence, which collectively shape how delivery workers negotiate risk, dignity, and resilience in the platform economy.

### Participants’ profile

The participant group comprised 10 food-delivery riders operating in urban and peri-urban areas of Tamil Nadu, India. The participants were between 24 and 35 years old, and although the majority were men, the sample included only one female rider. Their educational backgrounds ranged from secondary schooling to undergraduate degrees, with most riders discontinuing their formal education before entering the workforce. These platforms include Swiggy and Zomato, India’s two dominant food delivery services. Years of experience ranged from just over 1 to 4, with most participants working full-time and often exceeding standard labour hours.

Daily working hours varied between 8 and 13 hours, reflecting the intensity and time-bound nature of delivery. Monthly incomes fluctuated from ₹11,000 to ₹16,000, depending on location, delivery frequency, incentives, and penalty deductions. All the participants worked without formal employment contracts, accident insurance, or paid leave. They rely on personal smartphones and two-wheelers, typically without employer reimbursements for fuel or maintenance. These demographic and occupational conditions offer critical insights into the context of precarity, which structures participants' lived experiences. The following thematic analysis explores how these structural realities translate into emotional strain, physical fatigue, and social disconnection in everyday life.

Table I summarises the sociodemographic characteristics of the 10 participants. The table includes key variables such as age, gender, years of experience, daily working hours, monthly income, platform affiliation, and educational background. All the data were self-reported and presented using anonymized identifiers to protect confidentiality.

**Table I.** Participants’ profile.

Participant ID	Age	Gender	Platform	Experience (Yrs)	Daily hours	Monthly income (Rs.)	Education
P1	24	Male	Swiggy	1.5	10	12000	High School
P2	28	Male	Zomato	3	12	15000	Graduate
P3	32	Male	Swiggy	2	11	13000	High School
P4	26	Male	Zomato	1	9	11000	Secondary
P5	29	Male	Swiggy	2.5	13	16000	Graduate
P6	35	Male	Zomato	4	10	14000	High School
P7	30	Male	Swiggy	3	12	15000	Secondary
P8	27	Male	Swiggy	1.2	8	12500	High School
P9	31	Male	Zomato	2.8	11	14500	Graduate
P10	33	Female	Swiggy	2	9	13500	Secondary

Note: This table presents the basic demographic profile of the study participants, including age range, employment status, and length of experience in food-delivery work. Pseudonyms are used to protect participant anonymity.

### Thematic overview

Through a reflexive thematic analysis of in-depth interviews with 10 food delivery riders, 5 core themes emerged to illuminate the psychosocial, emotional, and physical complexities of their work experiences. These themes were not predefined, but emerged inductively through close engagement with participant narratives, iterative coding, and interpretive reflection. Each theme captures patterns of meaning that extend beyond individual cases, reflecting shared experiences of structural vulnerability, emotional burden, and symbolic exclusion. The analysis was informed by the conceptual framework of labour precarity, emotional labour, and symbolic violence, which provided a lens to interpret how riders internalise and navigate their working conditions. The themes are presented below in summary form and explored in detail in the subsequent sections. Table II provides a thematic summary of the five major identified themes.

**Table II.** Thematic summary.

Theme Title	Description	Theoretical Anchor	Illustrative Participant Insight
Surviving Precarity	Navigating unstable income, long hours, and financial fragility	Labour Precarity	*“Even after 12 hours, I barely cover expenses.”*
Embodied Exhaustion	Physical strain and chronic fatigue due to relentless delivery demands	Bio-political Labour	*“My body hurts every day… no time to rest.”*
Emotional Discipline Under Surveillance	Emotional self-regulation under rating pressure and customer scrutiny	Emotional Labour	*“Even when they scream, I must smile.”*
Internalised Struggles	Self-blame and normalisation of hardship due to internalised platform narratives	Symbolic Violence	*“I failed today—it’s my fault, not the app.”*
Fragmented Social Lives and Mental Fatigue	Isolation from family and erosion of social life due to overwork	Social Disconnection	*“I don't remember when I last had dinner with my family.”*

Note: This table summarises the five major themes identified through reflexive thematic analysis, capturing the psychosocial and embodied challenges faced by food-delivery riders in urban India.

### Theme 1: surviving precarity

This theme captures the economic and emotional strain experienced by food delivery workers as they navigate structurally unstable working conditions. Many riders were not only subject to irregular earnings or a lack of social protection but also in the midst of many micro-crises, which further fragmented their economic security, limited their autonomy, and inflicted mental fatigue. Customer behaviour, algorithmic opacity, and the systemic absence of institutional support often intensify these experiences.

Participants noted that income losses could occur due to errors beyond their control, including incorrect customer address information, miscommunication, and platform mismanagement. A rider delivered an order to a friend of the customer as per instructions, but was penalised when the platform deemed it incomplete.

*“I handed over the food and left. Later, I got a call from the company saying the customer didn’t receive the order. I tried to explain, but they closed it. I lost money for that order and the whole day’s earnings.”* (P8)

A lack of resources available to workers compounded the economic impact of such incidents. Their position as independent contractors meant that they bore the brunt of every miscommunication or system failure with no procedural safeguards. Precarity, in this sense, was not only economic but epistemic—riders did not always understand how or why they were being penalised, reinforcing their vulnerability to platform logic.

Social misunderstanding also contributes to emotional distress. Another participant recounted being misidentified and verbally confronted while attempting to complete delivery. The ambiguity of addresses and the pressure to deliver quickly place riders in socially awkward or hostile situations.

*“I thought that was the last house because they had ordered before. However, someone from the other house came out and accused me of staring at them. They thought I was checking them out. I felt so hurt. I stayed calm and just left.”* (P6)

This account illustrates how the precarity of gig work extends to the social realm, exposing riders to stigma, suspicion, and public misrecognition. Despite their essential role in the urban food economy, these incidents reinforced the feeling of being treated less.

Perhaps the most emotionally devastating accounts emerged when participants were not only unfairly blamed but also coerced into absorbing financial loss and verbal abuse. One rider described a scenario in which delayed food preparation in a restaurant leads to customer frustration. Despite explaining the situation, he was verbally attacked, threatened, and paid to avoid escalation.

*“The customer blamed me and told me to go back with the food. I couldn’t reach customer support. I just gave the money from my own pocket. He still shouted at me. I was mentally drained. That day, I really thought of quitting. I felt completely alone.”* (P3)

This narrative reflects not only the rider’s emotional exhaustion but also the internalised guilt and self-blame that can emerge when structural failures are perceived as personal shortcomings. Moreover, it highlights the emotional efforts riders must make to negotiate unpredictable emotional exchanges without institutional support.

Interpreted through the lens of labour precarity and symbolic violence (Bourdieu, 1991), these experiences suggest that riders not only endure material instability but also participate in a system that subtly compels them to accept exploitation as routine. Many participants spoke of “just needing to get through the day,” reflecting a normalisation of hardship. In the absence of formal employment protection, riders internalise the financial and emotional costs of systemic failure.

In conclusion, surviving precarity does not only mean survival in poor economic conditions. It also refers to survival in a mentally drained and structurally unprotected workplace. Furthermore, it entails being socially invisible. Although riders are not responsible for these issues, they still face their consequences. Further, they do not have the institutional power to challenge their marginalisation flexibility. This endurance, framed as resilience, masks the structural indifference that characterises platform-mediated labour in the Indian gig economy.

### Theme 2: embodied exhaustion

This theme reflects the cumulative physical and emotional toll borne by food delivery riders due to continuous, high-pressure work conditions and systemic inefficiencies. In this context, embodied exhaustion is more than just bodily fatigue; it includes the bodily and emotional effects of being blamed for delays and mistakes over which the rider has no control. The relentless pace of work combined with the physical strain of commuting in traffic, exposure to extreme weather, and tight delivery schedules results in both physiological and psychological weariness.

Many respondents spoke about how restaurant delays often led to customer frustration, which they found unfair. Despite riders' best efforts, they often face criticism and see their reputations damaged as customers start taking the blame for delivery delays. One participant described the demoralising effect of receiving abuse due to false updates generated by restaurant staff:

*“In restaurants, there are delays, but the app shows the food is ready. Customers think we are late or wasting time, and start scolding us. However, the delay is not our fault. Still, we are blamed. It’s so frustrating.”* (P7)

This scenario exemplifies the disjuncture between rider responsibility and system accountability. While platforms and restaurant staff share operational controls, they remain at a visible and vulnerable interface with customers. This imbalance intensifies the embodied stress of delivery work, as riders must not only manage time and logistics but also absorb emotional reactions triggered by failures outside their control.

Another participant shared an incident involving a mistaken food item, in which the customer, already agitated by the error, responded with verbal aggression that left lasting emotional marks.

*“The customer ordered a vegetarian pizza. I delivered it and left. Later, he called and kept scolding me in English. I explained calmly—it was the restaurant’s mistake—but he did not stop. He did not even apologise. It was really hurtful.”* (P4)

Here, exhaustion was not due solely to physical exertion but to the emotional labour of self-regulation. The rider had to remain composed, apologetic, and professional despite being the target of misplaced blame. Such episodes contribute to a state of chronic emotional depletion in which riders internalise faults and manage customer dissatisfaction with little or no support from platforms or restaurants.

A third rider explained that incomplete combo meals created further strain—not only because of customer complaints but also because they interfered with work targets and schedules. The app’s interface, which simplifies orders into generic “combos,” often fails to display individual items. The rider is vulnerable to disputes that cannot be anticipated or resolved independently.

*“Sometimes one item in a combo is missing, but we do not know what is inside. Some customers understand, but others refuse the delivery unless we go back. If the restaurant is far, it takes a lot of time and messes up our targets.”* (P1)

This account illustrates how systemic design flaws in the platform’s interface shift operational pressure onto riders, who are forced to manage the consequences of logistical breakdowns in real time. These incidents disrupt work rhythms, delay subsequent deliveries, and affect earnings. The physical effort required to return to distant restaurants, combined with the stress of navigating customer dissatisfaction, produces a cycle of fatigue that is difficult to recover from.

Taken together, these narratives reveal that embodied exhaustion in platform labour is not simply about working long hours or navigating traffic, but also about the bodily inscription of structural inefficiencies, platform opacity, and emotional asymmetry. Riders must constantly control their bodies, times, and emotions within systems that afford no rest or margin of error.

When we examine this situation through the concepts of bio-political labour and emotional regulation under platform surveillance (Lang et al., 2023; Wharton, 2009). We can see that riders’ exhaustion shows that their physical and emotional discipline is used to create a service model in which responsibility is displaced downwards. This system causes chronic depletion in which the body simultaneously becomes a site of accumulation, fatigue, frustration, and unrecognised endurance.

Ultimately, the lived experience of delivery riders reveals a striking paradox of platform work: while being branded as flexible and independent, the most quotidian impression is one of relentless demand, somatic wear, and invisible affective labour. The dynamics created are a form of slow harm to well-being. However, the state continues to be hidden behind technology or consumer efficiency.

### Theme 3: emotional discipline under surveillance

This theme reflects how food delivery riders internalise the need to regulate their emotions in response to frequent experiences of humiliation, exclusion, and symbolic devaluation in their everyday work environments. Although their efforts are highly visible to customers and essential to running the platform, riders feel socially invisible. They face disrespect from restaurant staff, customers, and their own family. To continue working, participants described the need to suppress emotional responses, such as anger, shame, and hurt, choosing instead to remain composed, silent, or “disciplined” in situations where they felt marginalised or dehumanised.

One rider described a recurring experience in which restaurants refused basic access to facilities, reinforcing the sense of being unwanted despite performing critical services.

*“Most restaurants and their staff, including the security, show very little or no respect towards us food-delivery riders. In some places, we’re not even given water. If we go inside, they tell us to wait outside until there’s a notification. They don’t care about us or our needs.”* (P3)

This account illustrates the emotional strain created by a systemic disregard. Although riders are required to wait for long hours and perform timely pickups, they are excluded from the spaces where they help sustain themselves. These boundaries are not just physical but also symbolic, reinforcing hierarchies between service providers and frontline gig workers. The need to maintain emotional control—to avoid confrontation or complaints —emerges not from personal restraint alone, but from a workplace culture that discourages riders from asserting basic dignity.

Another participant recounted how a routine payment interaction became a moment of quiet humiliation as a customer’s body language communicated discomfort and avoidance.

*“After delivering the order and collecting payment, they gave me the change like I was someone they didn’t want to touch. It really hurt. It feels like there is no respect for people like us in orange uniforms. It is like nobody values what we do.”* (P9)

Such small but significant moments accumulate across the rider’s working day. Although no explicit abuse was present, social cues, such as refusing physical proximity and withholding acknowledgement, conveyed a consistent message of inferiority. Riders often described this not with outrage, but with quiet resignation, suggesting that the emotional labour of maintaining composure in the face of such microaggressions is deeply ingrained.

The restrictions faced while waiting for food at pick-up points were another source of distress. Even when restaurants have designated areas for delivery personnel, riders often feel policed and monitored when attempting to access the basic amenities.

*“We riders have a separate place to pick up food, but if I go inside to get water, they question me—‘why are you here?’ It makes me feel really bad and disrespected.”* (P4)

This narrative points to a clear disconnect between riders’ roles in maintaining platform efficiency and how they are treated in the physical spaces of food delivery. Their presence is tolerated only to the extent that it serves the operational flow, and not as individuals with needs. The emotional toll of this treatment is not explosive but internalised, evident in how riders describe feeling “bad” or disrespected without confronting the systems that perpetuate such conditions.

Finally, one rider explained how the lack of respect extended beyond the workplace and into his personal relationships, as family and peers questioned the social legitimacy of his work:

*“Sometimes my family asks why I’m doing this kind of job. They say it is not respected. Even my friends in IT make fun of it. They think it’s not real work.”* (P10)

This statement underscores how emotional discipline extends into riders’ social lives, where they must manage not only external disrespect but also internal doubts about the value of their labour. Despite earning income and maintaining responsibility, they feel inadequate compared to their peers in more socially recognised professions.

Across these accounts, riders do not resist or challenge the disrespect they face. Instead, they absorbed it, maintained an outward calm state, and continued to perform their duties. Emotional regulation is not merely individual; it is embedded in the platform economy’s structure, which demands that workers remain polite, responsive, and efficient regardless of how they are treated. The results clearly show that riders’ ability to continue working depends not only on physical endurance but also on the constant, often painful effort to suppress emotional responses to devaluation.

### Theme 4: internalised struggles

This theme captures how food delivery riders absorb the pressures of platform-driven work into their personal identities and routines, often at the expense of emotional and social well-being. The riders’ narratives reveal a pattern of self-regulation and sacrifice, where financial hardship, unmet responsibilities, and physical exhaustion are normalised as part of being a “committed” worker. Rather than resisting these structural burdens, participants internalised the expectations placed upon them, modifying their behaviour, suppressing personal needs, and framing overwork as a necessary trade-off for survival and responsibility.

Several riders described the impossibility of maintaining a stable income if personal emergencies disrupted their weekly targets. In such cases, the economic model of gig work offers no flexibility, compelling them to borrow money or to skip essential life events. One participant reflected on the weight of familial responsibility and how it forced him to choose work over personal and social engagement.

*“At times, I can't meet my weekly targets due to personal issues that require me to take time off. Then I don’t earn enough, so I borrow money. Since my father passed away, I’m the only one supporting my family. My friends invite me out, but I usually say no. I even miss family functions. I have to work more to repay my loans and plan for the future.”* (P5)

This narrative illustrates how deeply personal the consequences of an economic interruption are. Participants accepted the need to forgo rest and social interaction, framing it as part of a larger moral obligation to provide for their families. In this way, the instability of gig work becomes routinized, with its consequences shouldered privately rather than structurally challenged.

Another rider shared that he works “day and night” without pause to ensure he meets financial commitments, describing how even the basic idea of leisure must be suspended:

*“I have to work day and night tirelessly to meet both my personal needs and support my family. To fulfil my commitments, I need to complete my targets. There’s no time for leisure. When I feel low, I talk to my friends on calls to share our experiences. That helps a bit.”* (P2)

Here, the rider acknowledges the emotional cost of constant work and hints at the collective coping mechanisms. However, the sense of internal responsibility dominates the narrative—he does not frame this routine as unjust or unsustainable but simply as necessary. Emotional burden is acknowledged but not externalised; instead, it is managed informally through peer support, reinforcing the deep embeddedness of this sense of personal obligation.

Many participants reported avoiding even important life events to prioritise work and maintain earnings continuity. One rider expressed regret about missing his own special occasions but felt unable to make different choices:

*“I can't go to parties or even my own special events like anniversaries, birthdays, or weddings because of my responsibilities. I want to be more responsible, so I put work first. My commitments make me run behind my job.”* (P6)

Another rider described the slow erosion of his social presence, noting that exhaustion and work schedules had displaced his ability to engage with others.

*“I don’t have much of a personal or social life. I start early and come back late. I’m always too tired to join in any conversation or activities. I just focus on completing my work.”* (P10)

These reflections illustrate how riders begin to view social withdrawal as a natural byproduct of their role. Work has gradually invaded time, space, and identity, thereby replacing community connections with isolation. The perceived urgency of the delivery schedules and performance targets displaced basic conversations with family or friends.

Finally, one participant spoke of how the cost of maintaining a delivery routine—fuel, repairs, and wear on his body—forced him to ration his participation in life outside work:

*“I avoid social gatherings unless absolutely necessary. If I take a day off, I lose progress for the whole week. I drive at least 50 kilometres every day. Petrol, servicing, punctures—it all adds up. I just can’t afford to go out often.”* (P1)

These narratives shared a striking sense of quiet compliance. Rather than challenging the conditions that demanded such sacrifices, the participants framed their struggles as personal shortcomings or necessary trade-offs. Financial instability, emotional fatigue, and social detachment were accepted not as structural injustices but as normal aspects of being a “or responsible” rider. Their strategies—working longer hours, withdrawing from social life, and internalising guilt—point to a deeper pattern in which overwork and under-recognition are woven into daily life.

### Theme 5: fragmented routines and physical deterioration

This theme conveys the gradual breakdown of food delivery riders’ physical stamina and daily functioning due to prolonged exposure to demanding, poorly regulated work conditions. The participants reported consistent bodily discomfort, sleep disruption, and health concerns closely linked to the physical nature of their tasks and the absence of structural safeguards. Pain, exhaustion, and neglect of personal health were not experienced as episodic challenges but as persistent aspects of everyday work life.

Several riders described how harsh weather conditions and pressure to avoid penalties compelled them to continue working despite being unwell or physically vulnerable. One participant was aware of his tendency to fall ill in the rain but was nevertheless pushed through delivery tasks to protect his income.

*“During rainy days, I work despite knowing that I easily catch colds and fevers due to my body condition. I have to deliver food regardless of heavy rain to avoid penalties. I’ve suffered a lot physically during that time. It leads to pain in my body and neck every day.”* (P7)

This narrative underscores how delivery riders are forced to ignore early signs of illness or discomfort to meet delivery timelines and avoid financial penalties. Rather than being offered support or flexibility during adverse conditions, the participants reported working through illness to remain economically afloat.

Another rider detailed how the tools required for the job, such as helmets, contributed to sustained physical discomfort, while prolonged sun exposure and erratic schedules further undermined basic health.

*“Wearing a helmet all day gives me neck pain. If I don’t wear it, my eyes hurt. Staying in the sun for too long causes body tanning, and not eating on time gives me stomach problems. Worst part is mosquito bites while waiting for food at restaurants.”* (P9)

The layering of such physical irritants from heat, hunger, and insect exposure reveals how riders’ bodies are continuously overexposed and undercared for. With no institutional mechanisms for rest, shade, or hygienic waiting areas, discomfort becomes routine and minor ailments endure without complaints or treatment.

Others have described the more serious consequences of repetitive motion, postural strain, and inadequate recovery time. One participant recounted how constant gear shifting and long riding hours left him with chronic joint and muscle pains.

*“Continuous work has caused pain in my shoulders. The bone in my right shoulder is scratched, and my left knee hurts from changing gears. I don’t even have time to use the restroom. I often get dehydrated, and sometimes I feel dizzy when riding in the sun. Eating outside food messes up my stomach.”* (P1)

These remarks suggest a cumulative form of physical decline in which untreated pain, disrupted routines, and dehydration become part of an accepted work rhythm. Such endurance is not framed as a health issue, but as part of the job’s demand, pointing to the normalisation of bodily neglect.

Another participant pointed to the effect of work-related anxiety on sleep and the bodily toll of insufficient rest:

*“One major issue I face is headaches from the heat, and I struggle to get proper sleep because I’m always thinking about targets. I often sleep irregularly and skip sleep if I haven’t met my quota. I also get back pain from all the long travel.”* (P2)

Fatigue is both physical and psychological, and the pressure to perform interferes with rest, recovery, and emotional regulation. Physical symptoms such as headaches, poor sleep, and back pain are tightly interwoven with mental strain.

Another rider noted the compounding effect of air pollution and road traffic on his body, particularly during prolonged rides.

*“I often get neck and back pain. When I ride behind lorries, dust gets into my eyes and causes redness, itching, and pain.”* (P8)

This account further highlights how the environments in which riders operate, such as polluted roads, heavy vehicles, and long-distance commuting, amplify the physical stress of their work. Eye problems and chronic aches are routinely treated as background noise, defined as exposure and repetition.

Collectively, these narratives depict a workforce whose physical and daily rhythms are shaped by exhaustion, overexposure, and a persistent deferral of care. The bodies of riders become the terrain where the pressures of the gig economy play out most visibly; riders are expected to deliver efficiency and punctuality, while absorbing the costs of pain, risk, and deprivation. Despite enduring constant bodily strain, the participants rarely framed their condition as a failure of the system. Instead, they described it as a necessary reality for their jobs, reinforcing the invisibility of their health sacrifices.

To visually summarise the analytical structure of the findings, Table III presents a thematic map illustrating the five major themes and their interrelations. These themes emerged through reflexive thematic analysis of the participant narratives and were conceptually anchored in the overarching constructs of labour precarity, emotional labour, and symbolic violence. This map reflects how riders' lived experiences are shaped by the intersecting material, emotional, and symbolic dimensions of platform-mediated work.

**Table III.** Thematic map of the lived experiences of food-delivery riders.

Dimension (Main Theme)	Pattern (Second Order Theme)	Sub-Pattern (First Order Theme)
**Surviving Precarity**	Customer-related Precarity	Inaccurate locations and rude customer behaviour (P8) Wrongful accusations and misunderstandings at delivery sites (P6) Unfair blame for late deliveries, threats, and lack of support (P3)
	Economic Insecurity	Losing money due to customer/company disputes (P8, P3)
	Emotional Impact	Mental distress and contemplation of quitting due to negative customer interactions (P3)
**Embodied Exhaustion**	Restaurant-related Strain	Delayed food preparation and false app updates leading to misplaced customer blame (P7) Restaurant mistakes causing customer anger (P4) Missing items in combo orders due to app limitations (P1)
	Physical Strain	Need to wait and inability to move to next order due to restaurant/customer issues (P1)
	Emotional Toll	Being unfairly held responsible for issues outside rider’s control (P7, P4)
**Emotional Discipline Under Surveillance**	Social Disrespect	Lack of respect from restaurant staff/security (P3, P4) Denied basic needs (water, entry) (P3, P4)
	Customer Disrespect	Customers handing over change disrespectfully (P9)
	Social Stigma	Family and friends devaluing delivery work (P10)
**Internalised Struggles**	Financial Pressure	Inability to meet targets leading to borrowing (P5) Sole breadwinner responsibilities (P5)
	Sacrifice of Personal/Social Life	Missing outings, family functions, personal events (P5, P2, P6, P10)
	Relentless Work Schedule	Working day and night to meet targets (P2, P10)
	Coping Mechanisms	Informal meetings with peers for emotional support (P2)
	Social Withdrawal	Avoiding social gatherings due to financial/work pressures (P1, P10)
	Financial Strain from Job Expenses	High travel costs, maintenance, and related financial challenges (P1)
**Fragmented Social Lives and Mental Fatigue**	Physical Health Issues	Working in harsh weather (rain, heat) causing illness (P7) Helmet use causing neck pain, eye issues (P9) Long hours causing body pain, dehydration, sleep loss (P1, P2, P9, P8)
	Mental Fatigue	Mental exhaustion from constant work and target pressure (P2)
	Occupational Hazards	Exposure to dust, mosquito bites, and lack of restroom access (P1, P8, P9)

Note: This Table illustrates the five core themes derived through reflexive thematic analysis and their conceptual linkage to labour precarity, emotional labour, and symbolic violence.

## Discussion

This study explored the lived experiences of food delivery riders in India, focusing on their emotional, physical, and social realities within the analytical framework of *labour precarity, emotional labour,* and *symbolic violence*. The findings reveal that an embodied form of structural vulnerability manifests not only on the economic side but also in psychosocial and symbolic forms, affecting the delivery rider’s sense of self, well-being, and social connections. Riders’ narratives expose a paradoxical world of digital employment, which promises flexibility and entrepreneurship to conceal the structures of control, endurance, and invisibility (Komarraju et al., 2021; Wood et al., 2018).

This interpretation is grounded in the three theoretical perspectives that underpin this study. The instability, invisibility, and endurance observed among riders reflect the structural insecurity central to *platform precariousness* (Vallas & Schor, 2020), while the internalisation of hardship and acceptance of unfair conditions resonate strongly with *symbolic violence* (Bourdieu, 1991). The persistent regulation of emotions in hostile situations exemplifies *emotional labour* (Hochschild, 2015), exposing that everyday interactions of food delivery riders are regulated by economic necessity and algorithmically enforced affective norms. Although platforms celebrate autonomy and independence, riders’ lived realities depend on opaque algorithms and constant emotional discipline (Chan, 2022; Yu et al., 2022). At the same time, recent European research shows that couriers often construct narratives of dignity and meaningfulness within precarious conditions, revealing a paradox between subjective work value and structural insecurity (Pieczka & Miszczyński, 2025). This depicts the masking of the unequal distribution of risks by normalising the precarity of work. This indicates a structural contradiction between the lived reality and corporate culture.

These findings corroborate prior research on surveillance and health care control (Krishna, 2020; Popan & Anaya-Boig, 2022; Rosenblat and Stark, 2016) while extending these insights through rich, first-hand accounts of emotional suppression, humiliation, and bodily neglect. Narratives are not only economically insecure but also emotionally exhausted and socially isolated, maintaining composure and politeness even under disrespect and strain (Lang et al., 2023).

The theme “*Surviving Precarity*” most directly aligns with *platform precariousness*, as riders operate under algorithmic management that produces unstable incomes, unpredictable hours, and heightened vulnerability. Participants described absorbing platform-level failures–order cancellations, income fluctuations, and lost bonuses—as personal shortcomings, illustrating internalised risk. This overlap between *platform precariousness* and *symbolic violence* demonstrates how economic instability becomes psychological, discourages resistance, and normalises inequality (Cruz & Gameiro, 2023).

The theme “*Embodied Exhaustion*” captures the corporeal consequences of gig work. This study reports that helmet use is continuous, leading to neck pain among riders. Exhaustion was exacerbated by dizziness from heat and by digestive issues associated with irregular meals. These results challenge the sanitised image of platform labour and its corporal costs, consistent with the findings of (Huang, 2021) and (Nguyen-Phuoc et al., 2023). This underscores the treatment of humans as instruments and the causal role of algorithmic capitalism.

Meeting customer expectations and algorithmic management require constant emotional regulation by riders. The theme “*Emotional Discipline under Surveillance”* examines this emotional regulation and substantiates (Hochschild, 2015) and (Bucher et al., 2020) on emotional labour. The monitoring of algorithmic feedback, coupled with continuous customer satisfaction, points to invisible but visible emotional labour. The riders often face disrespect and eventually fall into a kind of stigma, shaping how they are perceived both at work and beyond. These emotional expectations intersect with “*internal struggles*” whereby slow self-blame and guilt become moralised responses to exploitation. Riders often compare overstrain and exhaustion with personal discipline rather than systemic failure, reflecting the psychological dimensions (Cameron, 2024; Newlands, 2020).

The theme, “*Fragmented Routines and Physical Deterioration,*” underlines how economic survival is pursued at the cost of bodily integrity. This theme substantiates Benach et al., (2014) and Jalil et al., (2023), highlighting the compromise on proper rest, hydration, and meals to maintain a minimal income. This finding reveals a close link between economic survival and bodily compromise. Though the chronic pain, dehydration, and interrupted sleep were treated as harms, the riders consider them “normal” conditions of work. These findings exemplify the convergence of *platform precariousness and symbolic violence,* in which bodily decline is accepted and moralised as proof of endurance.

Collectively, the seven themes- Surviving Precarity, Embodied Exhaustion, Emotional Discipline under Surveillance, Internalised Struggles, Fragmented Routines, Physical Deterioration, and Social Disconnection- demonstrate that platform labour reshapes not only the economic sphere but also the psychosocial fabric of urban livelihoods. These vulnerabilities are inseparable from India’s entrenched hierarchies of class, caste, and migration, where structural disadvantages comprise algorithmic control. In line with (Bourdieu, 1991) The notion of symbolic violence, riders’ endurance in the face of long hours, low pay, and health risks, reflects an internalised acceptance of inequality reframed as perseverance. Accordingly, this study argues that food delivery labour must be recognised not merely as a labour market issue but also as a public health concern (Apouey et al., 2020; Lang et al., 2023).

This study extends the theory of gig work in three ways by integrating emotional labour, symbolic violence, and platform precariousness. First, *emotional labour* in platform economies works through algorithmic metrics that emphasise politeness and self-regulation (Kwan, 2024; Wharton, 2009). Second, *symbolic violence* manifests through ranking systems and feedback loops that lead workers to internalise blame for structural failings (Cruz & Gameiro, 2023; Komarraju et al., 2021). Finally, *platform precariousness* extends beyond economic instability to encompass enduring psychosocial erosion, emotional depletion, and physical exhaustion (Vallas & Schor, 2020). Together, these insights provide a theoretically integrated, worker-centred understanding of the intertwined emotional, structural, and symbolic mechanisms that sustain digital labour regimes in the Global South.

### Policy implications

The Platform work has emerged as a profession worldwide. In India, gig workers are considered a distinct labour category, but they are not included in social protection schemes. The riders are classified as “partners” rather than employees, leaving them outside the law and doing precarious jobs. In India, they are excluded from the benefits of employment welfare schemes and are unable to bargain because of a lack of sector-specific legislative safeguards (Fairwork India, 2024). Food delivery riders face vulnerabilities that require intervention from the government and platform providers. The need to establish a proper grievance redressal mechanism, transparent task allocation using algorithms, and financial safeguards should be properly addressed. Governments must extend social security coverage, enforce pay transparency, and introduce occupational health regulations to ensure minimum safety (Vallas & Schor, 2020). There should be a conducive environment for collective representation, which would further enhance workers’ accountability. Such measures are vital for reducing psychosocial risks and promoting sustainability in the digital service economy.

### Limitations and future research directions

The small sample size for the region limits the generalisability of the findings. Furthermore, the attention paid to rider narratives means that the seller's and consumer's viewpoints go unexamined. Nonetheless, the richness of qualitative insights and the focus on participants’ own meanings, which large-scale surveys tend to neglect, are noteworthy limitations. Scholars and future research should focus on the gendered and caste-based differentials in gig work experiences, as well as comparative studies across regions and platforms.

Although this study offers valuable insights into the lived experiences of food delivery riders in South India, it is not without limitations. The sample was small and region-specific, comprising 10 participants from a single urban district. Although this allowed for a deep, context-rich analysis, it limited the broader applicability of the findings to other geographic regions, platform types, and demographic groups. The absence of transgender and caste-diverse participants also restricts the study’s ability to account for the intersectional vulnerabilities in the gig economy. Moreover, the study focused exclusively on riders’ perspectives, omitting the views of platform operators, restaurant staff, and customers and stakeholders, whose interactions significantly shape riders' experiences. Additionally, as a cross-sectional study, it captures only one moment in time. It does not reflect how working conditions or psychosocial outcomes evolve in response to policy shifts, technological changes, or long-term occupational exposure. Future research could benefit from more inclusive, gender-sensitive, and intersectional designs that include riders with diverse castes and socioeconomic backgrounds. Comparative studies across different cities, regions, and platforms can further illuminate the structural patterns and context-specific challenges. Longitudinal or mixed-methods approaches would also be valuable for assessing how the psychosocial impacts of gig work develop over time and whether policy- or community-level interventions can mitigate these effects. Comparative perspectives from other Global South contexts, such as Bangladesh and Nepal, which share socio-economic and cultural parallels with the Indian gig economy, could also offer valuable benchmarks for understanding both convergence and divergence in platform labour experiences. While such cross-national analyses are beyond the scope of this regionally focused study, they present a promising avenue for future research aimed at countering the Global North-dominated framing of gig work debates. Expanding the lens to include public health and governance perspectives could deepen the understanding of how platform labour not only shapes individual well-being but also reflects broader societal shifts like work.

## Conclusion

This study advances the understanding of the psychosocial dimension of platform labour by revealing how food delivery riders in India experience and internalise precarity, emotional strain, and symbolic subordination. Through the integrated lens of emotional labour, symbolic violence, and platform precariousness, the findings demonstrate that gig work extends beyond economic instability to encompass deeper forms of emotional exhaustion, bodily deterioration, and moralised self-discipline. Riders’ narratives show how algorithmic management normalises exploitation while framing endurance as a personal virtue, thereby concealing the systematic inequalities underpinned by digital labour. The seven themes identified in this study highlight how structural, affective, and symbolic mechanisms converge to shape workers’ wellbeing and social identity in an expanding gig economy. Recognising these experiences as both labour and public health concerns underscores the urgent need for policy frameworks that extend legal, social, and psychological protection to platform workers. While the study’s small, context-specific sample limits generalisability, its depth offers critical insights into the lived realities of precarious work in the Global South. Ultimately, this research contributes to a more human-centred understanding of digital labour, urging scholars and policymakers alike to foreground dignity, health, and justice in the governance of emerging regimes.

## Data Availability

The data supporting the findings of this study are not publicly available due to the confidential nature of the interviews and the ethical commitment to protect participant anonymity. Data relevant to this analysis are available from the corresponding author upon request.
